# Co-administration of GnRH-agonist and hCG (double trigger) for final oocyte maturation increases the number of top-quality embryos in patients undergoing IVF/ICSI cycles

**DOI:** 10.1186/s13048-024-01465-6

**Published:** 2024-07-03

**Authors:** Binbin Tu, Hua Zhang, Lixue Chen, Rui Yang, Ping Liu, Rong Li, Jie Qiao

**Affiliations:** 1 Center for Reproductive Medicine, Department of Obstetrics and Gynecology, Peking University Third Hospital, Beijing, China; 2 National Clinical Research Center for Obstetrics and Gynecology, Peking University Third Hospital, Beijing, China; 3 Key Laboratory of Assisted Reproduction (Peking University), Ministry of Education, Beijing, China; 4 Beijing Key Laboratory of Reproductive Endocrinology and Assisted Reproductive Technology, Peking University Third Hospital, Beijing, China; 5 Research Center of Clinical Epidemiology, Peking University Third Hospital, Beijing, China

**Keywords:** GnRH-antagonist, Human chorionic gonadotropin, GnRH-agonist, Controlled ovarian hyperstimulation, Intracytoplasmic sperm injection, In vitro fertilization

## Abstract

**Background:**

The utilization of a double trigger, involving the co-administration of gonadotropin-releasing hormone agonist (GnRH-a) and human chorionic gonadotropin (hCG) for final oocyte maturation, is emerging as a novel approach in gonadotropin-releasing hormone antagonist (GnRH-ant) protocols during controlled ovarian hyperstimulation (COH). This protocol involves administering GnRH-a and hCG 40 and 34 h prior to ovum pick-up (OPU), respectively. This treatment modality has been implemented in patients with low/poor oocytes yield. This study aimed to determine whether the double trigger could improve the number of top-quality embryos (TQEs) in patients with fewer than three TQEs.

**Methods:**

The stimulation characteristics of 35 in vitro fertilization (IVF) cycles were analyzed. These cycles were triggered by the combination of hCG and GnRHa (double trigger cycles) and compared to the same patients’ previous IVF attempt, which utilized the hCG trigger (hCG trigger control cycles). The analysis involved cases who were admitted to our reproductive center between January 2018 and December 2022. In the hCG trigger control cycles, all 35 patients had fewer than three TQEs.

**Results:**

Patients who received the double trigger cycles yielded a significantly higher number of 2PN cleavage embryos (3.54 ± 3.37 vs. 2.11 ± 2.15, *P* = 0.025), TQEs ( 2.23 ± 2.05 vs. 0.89 ± 0.99, *P* < 0.001), and a simultaneously higher proportion of the number of cleavage stage embryos (53.87% ± 31.38% vs. 39.80% ± 29.60%, *P* = 0.043), 2PN cleavage stage embryos (43.89% ± 33.01% vs. 27.22% ± 27.13%, *P* = 0.014), and TQEs (27.05% ± 26.26% vs. 14.19% ± 19.76%, *P* = 0.019) to the number of oocytes retrieved compared with the hCG trigger control cycles, respectively. The double trigger cycles achieved higher rates of cumulative clinical pregnancy (20.00% vs. 2.86%, *P* = 0.031), cumulative persistent pregnancy (14.29% vs. 0%, *P* < 0.001), and cumulative live birth (14.29% vs. 0%, *P* < 0.001) per stimulation cycle compared with the hCG trigger control cycles.

**Conclusion:**

Co-administration of GnRH-agonist and hCG for final oocyte maturation, 40 and 34 h prior to OPU, respectively (double trigger) may be suggested as a valuable new regimen for treating patients with low TQE yield in previous hCG trigger IVF/intracytoplasmic sperm injection (ICSI) cycles.

## Background

The in vitro fertilization (IVF)/intracytoplasmic sperm injection (ICSI) is a widely used technique for infertility treatment. The gonadotropin-releasing hormone (GnRH) antagonist protocol is a frequently utilized regimen during controlled ovarian hyperstimulation (COH) for IVF. GnRH antagonists (GnRH-ant) competitively inhibit GnRH receptors, thereby suppressing gonadotropin release and preventing a premature luteinizing hormone (LH) surge. The advantages of the GnRH-ant protocol include the absence of a fare-up effect, rapid decline in LH and follicle-stimulating hormone (FSH) levels, a lower gonadotropin dose, and shorter duration of ovulation stimulation [1, 2].

With the steady expansion of GnRH-ant usage in clinical practice, GnRH-a was introduced to promote final oocyte maturation [3]. GnRH-a induce a surge of endogenous LH and FSH, similar to the preovulatory gonadotropin surge in natural cycles [4]. However, compared with the traditional human chorionic gonadotropin (hCG) trigger, using a GnRH-a alone triggers a shorter LH surge, resulting in luteal phase dysfunction. This dysfunction necessitates intensive luteal phase support to improve pregnancy outcomes [5]. Therefore, hCG, GnRH-a, or their combination (“dual” or “double” trigger) can be utilized for final oocyte maturation. Among them, dual trigger, simultaneous utilization of hCG and GnRH-a 36–38 h before ovum pick-up (OPU), has exhibited to enhance oocyte maturation and improve the number of top-quality embryos (TQEs), pregnancy rates, and live birth rates in individuals with ovarian hypo-responsiveness or normal ovarian responsiveness [6–10].

Another method involving the utilization of GnRH-a and hCG for final oocyte maturation, administered 40 and 34 h prior to OPU respectively, is known as the double trigger. This approach was initially used in patients with empty follicle syndrome (EFS), despite having apparently normal follicular development and estradiol levels [11]. Therefore, it also improved the cycle outcomes for patients with a low proportion of mature oocytes or a low number of oocytes retrieved per number of preovulatory follicles in previous 12 and 8 hCG-trigger cycles, respectively [12, 13]. However, in cases who have undergone a previous hCG trigger cycle, the benefits of the double trigger on oocyte retrieval, MII oocyte yield, TQEs, and final reproductive outcomes remain elusive.

In the present study, the double trigger was utilized in patients who underwent the GnRH-ant COH protocol and had fewer than three TQEs in a previous hCG-trigger cycle. The aim was to investigate whether there were differences in the number of oocytes retrieved, the number of embryos formed, and pregnancy outcomes.

## Methods

### Study participants

All patients with fewer than three TQEs who received hCG trigger for final oocyte maturation between January 2018 and December 2022 in the Reproductive Center of Peking University Third Hospital (Beijing, China) were evaluated. Among them, only those who received a double trigger (GnRH-a and hCG, 40 and 34 h prior to OPU, respectively) in the subsequent IVF cycle for final oocyte maturation within one year were included. All patients underwent the GnRH-ant COH protocol in both IVF cycles. The study was approved by the Institutional Research Board of Peking University Third Hospital (Approval No. 2018SZ-002).

### Ovarian stimulation and IVF procedures

In both cycles, ovulation induction was performed by the administration of recombinant FSH at the 2nd or 3rd days of the menstrual cycle, with the primary dose of 112.5IU ~ 225IU in each patient. A GnRH-ant (Cetrotide, Merck-Serono) was administered when a dominant follicle was found on ultrasound (mean diameter > 13 mm) or when estradiol level was ≥ 1,000 pmol/L, at a dose of 0.25 mg/day. This flexible antagonist protocol was continued throughout ovarian stimulation. Gonadotropin dose was further adjusted according to serum estradiol level and follicular diameter measured by vaginal ultrasound, which was conducted every 2 or 3 days. When at least two follicles reached 18 mm or three follicles reached 17 mm in diameter, final oocyte maturation was triggered by either: (1) In the first IVF cycles (control cycles), recombinant hCG (Choriogonadotropin alfa, Ovidrel 250 µg, Serono) was administered 36–38 h prior to oocyte pickup (OPU); or (2) In subsequent IVF cycles (study cycles), a combination of GnRH-a (Triptorelin acetate, Dophereline 0.2 mg, Ipsen-Biotech, Inc., Paris, France) and recombinant hCG (250 µg) was administered 40 and 34 h prior to OPU, respectively.

Transvaginal ultrasonography-guided oocyte retrieval was carried out after final oocyte maturation. Routine IVF or ICSI was then performed, as appropriate. A maximum of two embryos were transferred in fresh or frozen-thawed cycles, followed by luteal support with progesterone. Morphologically, TQEs were defined as those with six or more blastomeres on day 3, equally sized blastomeres and < 20% fragmentation, based on the individual embryo scoring parameters according to previously described definitions [14]. All other embryos were considered as poor-quality embryos.

### Pregnancy outcomes

hCG positivity was defined as serum β-hCG level higher than 5U/L measured 2 weeks after embryo transfer. Clinical pregnancy was defined as the detection of at least one gestational sac by ultrasonography. Ongoing pregnancy was defined as the visualization of an intrauterine sac with an embryonic pole, demonstrating cardiac activity after 10–12 gestational weeks. Live birth was defined as the delivery of live babies beyond 28 weeks of pregnancy or with a birth weight greater than 1000 g.

### Statistical analysis

Statistical analysis was carried out using SPSS 27.0 software (IBM Corp., Armonk, NY, USA). Normal distribution of data was assessed using the Shapiro–Wilk test. Data wereare presented as mean ± standard deviation (SD) or median (25th, 75th). The McNemar test, paired-samples t-test, and Wilcoxon signed-rank test, as appropriate, were utilized for making group-based comparisons. *P* < 0.05 was considered statistically significant.

## Results

### The basic characteristics of the study population

Totally, 35 patients were evaluated, and their demographic and clinical characteristics after the hCG trigger control cycle are presented in Table 1. Patients’ age and body mass index were 34 (32, 37) years and 21.90 (20.10, 24.40) kg/m^2^, respectively. Anti-müllerian hormone (AMH) level was 1.67 (1.20, 2.65) ng/ml, and the mean antral follicle count (AFC) was 7.50 (5.00, 11.75). The duration of infertility was 5 (4, 7) years. The number of previous pregnancies was 0 (0, 1). Of the participants, 22 (62.86%) patients had primary infertility, and 13 (37.14%) patients had secondary infertility. The causes of infertility included: ovulation factor in 12 cases (34.29%), tubal factor in 10 cases (28.57%), Endometriosis in 4 cases (11.43%), male factor in 3 cases (8.57%), and other factors in 6 cases (17.14%). The number of previously failed stimulation cycles was 2(1, 2).

**Table 1:** Baseline clinical and demographic characteristics of patients after hCG trigger cycle

	n = 35
Age (years)	34 (32, 37)
Body mass index (kg/m^2^)	21.90 (20.10, 24.40)
Anti-Mullerian hormone (ng/ml)	1.67 (1.20, 2.65)
Antral follicle count	7.50 (5.00, 11.75)
Duration of infertility (year)	5(4, 7)
No. of previous pregnancies	0 (0, 1)
Type of infertility	
Primary infertility	22 (62.86)
Secondary infertility	13 (37.14)
Cause of infertility	
Ovulation factor	12 (34.29)
Tubal factor	10 (28.57)
Endometriosis	4 (11.43)
Male factor	3 (8.57)
Others	6 (17.14)
No. of previous failure stimulation cycles	2 (1, 2)

Values were presented as median ± IQR or number (%)

### The characteristics and outcomes of stimulation cycles

The clinical characteristics of the hCG trigger control cycles and the double trigger study cycles are presented in Table 2. Expectedly, no significant differences were found between the two cycles in the length of stimulation (9.76 ± 1.21 vs. 9.32 ± 1.45 days, *P* = 0.092), dosage of gonadotropin (2601.84 ± 903.12 vs. 2770.74 ± 885.15 IU, *P* = 0.304), and peak estradiol on the day of hCG administration (8632.34 ± 5126.10 vs. 9214.84 ± 6502.59 pmol/l, *P* = 0.658). Moreover, 42.86% (15/35) of patients in the hCG trigger control group underwent routine IVF, and comparably, 22.86% (8/35) of patients underwent routine IVF in the subsequent double trigger cycle.

**Table 2:** Comparison between IVF cycles with double trigger (GnRH-a + hCG) versus hCG trigger

	hCG trigger cycle	Double trigger cycle	t value	P
Length of stimulation (day)	9.76 ± 1.21	9.32 ± 1.45	-1.737	0.092
Dosage of gonadotropin (IU)	2601.84 ± 903.12	2770.74 ± 885.15	1.045	0.304
Peak estradiol levels on day of hCG administration (pmol/l)	8632.34 ± 5126.10	9214.84 ± 6502.59	0.447	0.658
Fertilization method				0.065
Routine IVF (%)	42.86 (15)	22.86 (8)		
ICSI (%)	57.14 (20)	77.14 (27)		
Number of oocytes retrieved	9.66 ± 6.09	9.11 ± 6.30	-0.654	0.517
Number of MII oocytes (ICSI cycle)	4.43 ± 2.74	6.21 ± 3.93	1.516	0.153
Number of 2PN zygotes	2.71 ± 2.59	3.57 ± 3.39	1.432	0.161
Number of cleavage embryos	3.63 ± 3.81	4.40 ± 3.52	1.103	0.278
Number of 2PN cleavage embryos	2.11 ± 2.15	3.54 ± 3.37	2.35	0.025
Number of top-quality embryos	0.89 ± 0.99	2.23 ± 2.05	3.687	< 0.001
Proportion of MII per number of oocytes retrieved (%) (ICSI cycle)	59.27 ± 30.56	66.27 ± 27.97	0.985	0.342
Proportion of 2PN zygote per number of oocytes retrieved (%)	33.21 ± 27.56	44.22 ± 33.18	1.885	0.068
Proportion of cleavage embryo per number of oocytes retrieved (%)	39.80 ± 29.60	53.87 ± 31.38	2.11	0.043
Proportion of 2PN cleavage embryo per number of oocytes retrieved (%)	27.22 ± 27.13	43.89 ± 33.01	2.583	0.014
Proportion of top-quality embryo per number of oocytes retrieved (%)	14.19 ± 19.76	27.05 ± 26.26	2.463	0.019
Cumulative hCG positive rate per stimulation cycle (%)	8.57 (3/35)	20.00 (7/35)		0.289
Cumulative clinical pregnancy rate per stimulation cycle (%)	2.86 (1/35)	20.00 (7/35)		0.031
Cumulative persistent pregnancy rate per stimulation cycle (%)	0 (0/35)	14.29 (5/35)		< 0.001
Cumulative live birth rate per stimulation cycle (%)	0 (0/35)	14.29 (5/35)		< 0.001

Values are presented as mean ± standard deviation or percentage. The classified data was analyzed by McNemar test

The outcomes of the stimulation cycles are presented in Table 2. The number of oocytes retrieved (9.66 ± 6.09 vs. 9.11 ± 6.30, *P* = 0.517), MII oocytes (oocytes planned for ICSI) (4.43 ± 2.74 vs. 6.21 ± 3.93, *P* = 0.153), 2PN zygotes (2.71 ± 2.59 vs. 3.57 ± 3.39, *P* = 0.161), and cleavage stage embryos (3.63 ± 3.81 vs. 4.40 ± 3.52, *P* = 0.278) were comparable between the hCG trigger control cycles and subsequent double trigger study cycles. The proportions of MII oocytes (59.27 ± 30.56% vs. 66.27 ± 27.97%, *P* = 0.342) and 2PN zygotes (33.21 ± 27.56% vs. 44.22 ± 33.18%, *P* = 0.068) relative to the number of oocytes retrieved were comparable between the two subsequent cycles. However, double trigger study cycles resulted in a significantly higher number of 2PN cleavage stage embryos (3.54 ± 3.37 vs. 2.11 ± 2.15, *P* = 0.025) and TQEs (2.23 ± 2.05 vs. 0.89 ± 0.99, *P* < 0.001) compared with hCG trigger control cycles. Additionally, double trigger cycles yielded a significantly higher proportion of cleavage stage embryos (53.87 ± 31.38% vs. 39.80 ± 29.60%, *P* = 0.043), 2PN cleavage stage embryos (43.89 ± 33.01% vs. 27.22 ± 27.13%, *P* = 0.014), and TQEs (27.05 ± 26.26% vs. 14.19 ± 19.76%, *P* = 0.019) relative to the number of oocytes retrieved. Notably, in the hCG trigger control cycles, 16 patients had no TQEs, whereas in the double trigger cycles, 10 patients had no TQEs.

### Pregnancy outcomes

As no fresh embryonic transfer was performed in each patient within the two consecutive cycles, which could be attributed to the lack of transferable embryo or other reasons, no matching analysis of pregnancy outcomes in fresh cycles was conducted. The hCG positive rate, clinical pregnancy rate, ongoing pregnancy rate, and live birth rate in fresh transfer cycles were 21.1% (4/19) vs. 21.4% (3/14), 21.1% (4/19) vs. 7.1% (1/14), 10.5% (2/19) vs. 0% (0/14), and 10.5% (2/19) vs. 0% (0/14), respectively, in double trigger study cycles and hCG trigger control cycles. Furthermore, the cumulative pregnancy outcomes, including fresh embryonic transfer and frozen-thawed embryonic transfer per stimulation cycle were assessed (Table 2). The double trigger study cycles achieved higher cumulative clinical pregnancy rate (20.00% vs. 2.86%, *P* = 0.031), cumulative persistent pregnancy rate (14.29% vs. 0%, *P* < 0.001), and cumulative live birth rate (14.29% vs. 0%, *P* < 0.001) per stimulation cycle compared with the hCG trigger control cycles.

## Discussion

The present retrospective cohort study was conducted to assess the influences of the trigger of final oocyte maturation in GnRH-ant protocol cycles on patients who yielded fewer than three TQEs. According to the results, double trigger, the co-administration of GnRH-a and hCG for final oocyte maturation 40 and 34 h prior to OPU, respectively, to patients with less than three TQEs resulted in a significantly higher number of 2PN cleavage embryos and TQEs. Additionally, there was a higher proportion of cleavage embryos, 2PN cleavage embryos, and TQEs per number of oocytes retrieved compared with the hCG trigger control cycles. Meanwhile, the double trigger study cycles also improved cumulative clinical pregnancy rates, cumulative persistent pregnancy rates, and cumulative live birth rates per stimulation cycle compared with hCG trigger control cycles. Although the total number and proportion of MII oocytes were greater in the double trigger study cycles than those in hCG trigger control cycles, no significant difference was found between the two cycles. The variability in results of the present study may arise from the fact that not all patients underwent ICSI, which prevented the determination of the exact count of MII oocytes per patient. As the number of TQEs can be influenced by the quantity of MII oocytes, the significant increase in the number and proportion of embryos and TQEs found in the double trigger cycles suggests a higher count and proportion of MII oocytes in those cycles.

The results of the present study aligned with the results of several previous studies, in which a double trigger was superior to an hCG trigger. According to recently reported findings, the “double trigger” approach had been successfully used in a patient with recurrent EFS and finally delivered a healthy boy at gestation age of 38 weeks [11]. Similar outcome was observed in a patient with borderline form of EFS [15]. Meanwhile, the “double trigger” approach has exhibited a significant improvement in oocyte yield, the proportion of MII oocytes, and transferable embryos among patients with a low or poor yield of oocytes, or a low rate of mature oocytes (less than 50%), despite apparently normal follicular development and E2 level on the day of hCG administration. However, the impact on pregnancy rates remains inconclusive [12, 13]. The two aforementioned studies were both self-paired and involved small sample sizes of 8 and 12 patients, respectively, from the same research group. In 2019, the same researchers conducted a small-sample prospective randomized controlled study that further supported the conclusion. They found that poor responders in the double trigger group (n = 12) had a significantly higher number of TQEs compared with the hCG trigger group (n = 11) and the GnRH-a trigger group (n = 10) (1.1 ± 0.9 vs. 0.3 ± 0.8 vs. 0.5 ± 0.7) [16].

In the present study, the double trigger study cycles improved cumulative clinical pregnancy rates, cumulative persistent pregnancy rates, and cumulative live birth rates per stimulation cycle compared with hCG trigger control cycles. It may be due to more TQEs in double trigger cycles than in hCG trigger cycles. Obtaining higher quality embryos is critical for IVF-ET success, which is necessary for achieving a live birth. An appropriate trigger method is vital for oocyte maturation and TQE formation. The “double trigger”, consisting of the co-administration of GnRH-a and hCG for final oocyte maturation 40 and 34 h prior to OPU, respectively, differs from the ‘‘hCG trigger’’ by the addition of GnRH-a trigger, as well as the additional prolongation of the time between ovulation triggering and OPU. The abovementioned variations may be related to the prior outcomes of the double-trigger cycles.

During natural cycles, both LH and FSH surges are necessary to trigger ovulation. However, the routine hCG trigger lacks FSH receptor activity, and therefore does not fully resemble natural oocyte maturation and ovulation [17]. Unlike hCG, a GnRH-a trigger results in both LH and FSH surges [18], making it more similar to natural ovulation. The role of the natural mid-cycle FSH surge remains elusive. Both animal and human studies have indicated that FSH plays a role in ovulation and oocyte maturation. It stimulates the expansion of cumulus cells surrounding the oocyte and inducing the formation of LH receptors on luteinized granulosa cells, thereby increasing the likelihood of retrieving more MII oocytes [17, 19]. Expansion and dispersion of the cumulus cells enables the oocyte-cumulus cell mass to detach from the follicular wall before ovulation. In addition, a GnRHa trigger can activate the GnRH receptors on granulosa cells, regulating ovulation [20].

Prolonging the interval between ovulation triggering and OPU may also contribute to the positive outcomes in the double trigger cycles. In a natural cycle, resumption of meiosis and follicular rupture begin 18 and 34–36 h after the onset of the LH surge, respectively. Similarly, administering exogenous hCG causes follicular rupture after 37 h. LH concentration should be maintained above a threshold for 14–27 h to maximize oocyte maturation. Thus, follicular rupture and oocyte maturation are time-dependent processes, and the duration varies among diverse patients. It might be hypothesized that the process of cumulus expansion, facilitating the detachment of the oocyte from the follicular wall, may also require longer periods in some patients.

Noteworthy, dual trigger, simultaneous utilization of hCG and GnRH-a 36–38 h before OPU, has exhibited to enhance oocyte maturation and increase the number of TQEs in hypo-responders or normal-responders [6, 9]. Similar results were obtained by a systematic review and meta-analysis of randomized trials, and there may be an increase in clinical pregnancy rate in the dual trigger group in hypo-responders [21]. However, in opposite, prior research found no significant differences in the number of available embryos, and rates of fertilization, implantation, clinical pregnancy, and miscarriage between the dual trigger and hCG trigger in hypo-responders [22]. Whether the dual trigger improves pregnancy outcomes in normal-responders remains controversial. Some retrospective trials found no significant difference in clinical pregnancy rate [23, 24], contradicting the findings of a recent meta-analysis, in which the dual trigger group exhibited an increase in clinical pregnancy and live-birth rates [25]. It was broadly accepted that the selection of an individualized trigger is crucial for better pregnancy outcomes in different populations. In the present study, 35 patients were treated by the double trigger approach, and the majority of them were normal responders. It was found that the double trigger could improve the quantity and quality of embryos and pregnancy outcomes compared with the hCG trigger, which were consistent with the findings of previous small-scale studies [12, 13, 16]. The main difference between the dual trigger and the double trigger is the administration time of GnRHa. GnRHa is applied 36–38 h before OPU in the dual trigger, while it is administered 40 h before OPU in the double trigger. Additional prolongation of the time between trigger onset and OPU may contribute to greater outcomes because of variations in the time needed from hCG exposure to maturation of oocyte-cumulus complexes [26], and some patients may require longer triggering time for oocyte maturation and follicular rupture. In some cases, where no oocytes are aspirated from one ovary and hCG level is low, readministering hCG and aspirating the second ovary [27] or even re-aspirating the same follicles [28] have been suggested. However, whether the double trigger is superior to the dual trigger needs further investigation, as there are no comparative studies between the two trigger regimens.

To date, only 5 clinical research studies on the double trigger approach have been identified in PubMed, as previously mentioned. Among them, 2 were case reports, 2 were self-paired cohort historical studies, and one was a pilot prospective randomized controlled study. The sample sizes in these studies were relatively small, typically around 10 patients per group. Alongside the current study involving 35 patients, these 6 studies collectively suggested that the double trigger approach could enhance both the quantity and quality of oocytes and embryos, potentially leading to favorable pregnancy outcomes. Despite the limitations inherent in retrospective design and small sample size, the present study lays the groundwork for future large-scale, prospective, and randomized controlled trials aimed at a more comprehensive evaluation of the double trigger regimen. Furthermore, the self-paired nature of the present study ensured excellent baseline comparability of subject characteristics. However, given that the intervention was not administered simultaneously, the influence of time and the subsequent effects of previous interventions should be carefully considered.

## Conclusions

In conclusion, the double trigger, the combined usage of GnRH-a and hCG for final oocyte maturation, administered 40 and 34 h before oocyte retrieval, respectively, represents a potentially valuable new regimen in the treatment of patients with low TQE yield in previous hCG trigger IVF/ICSI cycles.

## Data Availability

No datasets were generated or analysed during the current study.
